# Validation of Masimo Pronto 7 and HemoCue 201 for hemoglobin determination in children from 1 to 5 years of age

**DOI:** 10.1371/journal.pone.0170990

**Published:** 2017-02-07

**Authors:** Teresa Shamah Levy, Ignacio Méndez-Gómez-Humarán, María del Carmen Morales Ruán, Brenda Martinez Tapia, Salvador Villalpando Hernández, Mauricio Hernández Ávila

**Affiliations:** 1 National Public Health Institute (INSP), Cuernavaca, Morelos, Mexico; 2 Center for Research in Mathematics, Aguascalientes, Aguascalientes, Mexico; Emory University/Georgia Institute of Technology, UNITED STATES

## Abstract

**Objective:**

To evaluate the accuracy and precision of HemoCue 201 (HemoCue) and Masimo Pronto 7 (Masimo) devices for measuring hemoglobin (Hb) in epidemiological studies, having venous blood samples as a gold standard.

**Material and methods:**

We measured Hb concentrations in a field sample of 148 children from one to five years of age. Masimo and HemoCue were used for capillary blood samples and an automatic analyzer for venous blood samples. Regression models with no intercept were constructed to measure precision and predictability, concordance correlations to measure accuracy and precision, and Bland-Altman limits of agreement as well as hierarchical linear models to estimate variance.

**Results:**

Both HemoCue and Masimo underestimated Hb concentrations compared to the gold standard. They respectively yielded the following results: regression coefficients of 0.887 and 0.876 with 98.7% and 98.6% predictability; concordance correlation coefficients of 0.183 (p<0.001) and 0.166 (p<0.001); and Bland-Altman variances of -1.51 and -1.62. With regard to Masimo specifically, the three-level Hierarchical Linear Model showed that 57.9% of total variance stemmed from random errors in repeated measures from the same subject.

**Conclusions:**

HemoCue and Masimo measure lower Hb concentrations than the gold standard. Their accuracy and precision levels are comparable. It is essential to ensure proper use of devices through enhanced training of field workers.

## Introduction

Anemia is the leading nutritional problem faced by public health worldwide. Of the 1.62 billion affected individuals, children under five years bear the heaviest burden with a prevalence of 47%. Anemia exerts a negative short-term impact on physical growth and cognitive development. In the long run, it undermines work productivity in adults with substantial economic consequences. [1, 2]

Anemia prevention and control strategies include improved dietary intake; food diversification, fortification and supplementation with iron and other micronutrients; targeted disease control; and education. [2]

In Mexico, prevalence of anemia reached 23.3% in 2012. [3] The government has responded by creating assistance programs to develop the capacities of beneficiary children through improved nutrition, money transfers for food items, access to low-cost milk fortified with micronutrients, school breakfasts for malnourished children in preschool facilities, health services and the promotion of healthy diets. [4]

As a means for evaluating the effectiveness of food assistance programs and characterizing anemia at baseline and follow-up measurements, there is growing interest in obtaining biochemical indicators for anemia at the population level. Accurate assessment of anemia levels in the population is indispensable for social programs to focus on the most vulnerable populations and evaluate the impact of interventions.

The international diagnostic indicator for anemia is blood hemoglobin (Hb) concentration, controlling for age, sex, altitude and physiological status. In children, anemia is defined as an Hb concentration of <11.0 g/dL. [2, 5] According to the International Committee for Standardization in Hematology, [6, 7] the determination of Hb with an automated analyzer and in a clinical laboratory is the gold standard for Hb measurement. However, the complexity of collecting and preserving venous blood samples during epidemiological studies has prompted the development of other methods for establishing adequate Hb concentration estimates.

Among the various portable devices developed for Hb measurement in the field, HemoCue, a method requiring 10 μl of capillary blood, is the one most extensively used.[3]

The HemoCue device has been found to offer high precision and accuracy in laboratory settings; [8, 9] however, mixed opinions have been expressed regarding its performance in the field. [10] For instance, Chen et al. [11] compared the precision and accuracy of HemoCue vs. a reference method for the assessment of Hb in venous, arterial, and capillary blood samples, and found that the former were considerably lower, specifically in capillary blood samples.

Masimo, another Hb measurement instrument currently available in the market, employs noninvasive technology (invasive procedures are those that penetrate or “invade” the body with a needle, tube, device or an endoscope), and measures arterial oxygen saturation (SpO2), pulse (PR), perfusion index (IP) and total Hb concentration (SpHb). Despite its fairly recent development (in 2008) and lack of available evidence validating its use in field studies, Masimo is the most commonly employed method in clinical practice.

Hiscock et al. [12] reviewed 39 publications and compared the Hb values of the Masimo co-oximeters (Rad-7™ and Pronto-7™) and HemoCue photometers (201) against the laboratory gold standard. Their results indicated that Masimo and HemoCue diverged from the standard by mean variances of -0.03 g/dL (95% CI -3.0, 2.9 g/dL) and 0.08 g/dL (95% CI -1.3, 1.4 g/dL), respectively. While both methods appeared to provide unbiased estimates, Masimo showed less precision and greater distribution in measurement than HemoCue.

Of the three available studies with children, only one evaluated Masimo. It sampled children under three in a clinical environment in Japan and used the Bland-Altman limits of agreement method to compare the capillary blood measurements of the Masimo and Microsemi® LC-667CRP devices. Resulting limits ranged from -2.76 to 1.56 g/dL. [13] All other studies have been conducted with adults and in clinical environments. [14, 15, 16]

Previous population studies suggest that HemoCue capillary Hb values are lower than venous Hb values. They also indicate that HemoCue Hb values are reproducible in venous—not capillary—samples. [17].

The objective of this study consisted in evaluating the accuracy and precision of the HemoCue and Masimo methods for Hb determination in epidemiological studies with children from one to five years (the age group most frequently selected for public health evaluations), having venous blood measures as the gold standard.

## Materials and methods

A pilot field study was conducted to validate the precision and accuracy of Hb measurement using HemoCue and Masimo in epidemiological studies. A sample size of 150 subjects was calculated to compare the HemoCue and Masimo measures with those of the gold standard, considering a confidence level of 95%, an error limit of 0.23 g/dL for mean Hb estimation and a standard deviation of 1.42 g/dL.

We assembled a sample of 148 children from one to five years of age who had participated in a study conducted to assess the impact of food assistance on the nutritional status of children in San Luis Potosí (Teresa Shamah Levy, Salvador Villalpando Hernández, Lucía Cuevas Nasu, Ignacio Méndez Gómez-Humarán; Elsa Berenice Gaona Pineda, Juan A. Rivera Dommarco. *“Evaluación del impacto de los programas de apoyo alimentario en el estado de nutrición de la población infantil del Estado de San Luis Potosí”*. 2013–2015 Project.FMSLP-2013-C02-208475 during August-October 2015). Hb measurements were obtained from participants using Masimo Pronto 7 (Masimo, Hannover, Germany), HemoCue 201 (HemoCue Inc., Mission Viejo, CA, USA) and the gold standard.

For an optimal reading with Masimo, each participant was sampled at rest, sitting, with the device positioned on a flat surface at the level of his or her hand. The ring sensor was placed on the left middle finger and the reading was obtained following the manufacturer directions.[8] Three measurements were taken at ten-minute intervals and the mean was used as the determination value.

The Masimo sensor collects the patient's vital signs and sends them to the device which, in turn, displays the calculated SpHb value in g/dL. The basic operation principle of this instrument is the differential absorption of multiple wavelengths of visible light (using spectrophotometry) to distinguish between oxyhemoglobin (oxygenated blood), deoxyhemoglobin (non-oxygenated blood), carboxyhemoglobin (carbon monoxide blood), methemoglobin (blood with oxidized hemoglobin) and other constituents of blood plasma. The amount of blood in the tissue fluctuates with the patient’s pulse (photo-plethysmography) and therefore changes the amount of light absorbed. Data are obtained by sending several infrared light beams (500 to 1300nm) through the capillary surface at the tip of the patient’s finger and measuring the changes in light absorption during the pulsatile blood cycle. The maximum radiant power of the strongest light beam is ≤ 25mW. The detector receives the light, turns it into an electronic signal and sends it to the instrument to perform the calculations. The signal from the sensor is transformed by Masimo Rainbow SET technology to calculate oxygen saturation, pulse and total Hb concentration (SpHb [g/dL]) in the patient. SpHb measurement relies on a calibration equation of multiple wavelengths which quantifies the percentage of total blood Hb. [18]

For HemoCue measurements, one capillary blood sample was taken from each subject and analyzed in the device. Repeated measurements (requiring finger punctures) were not viable; hurting children three times with an invasive procedure was unjustifiable. To operate the device, the user deposits the blood sample in a cuvette containing a dry reagent (isodioazide). Activated by the blood, the reagent ruptures the red blood cell membranes and releases the Hb content. Methemoglobin is formed in the cuvette by converting ferric iron to ferrous iron which, combined with azide, forms azide-methemoglobin. Absorbance is read photometrically at dual wavelength (565 nm and 880 nm) to control for turbidity. HemoCue converts the readings into Hb concentrations and displays them digitally as g/dL. [19]

For venous blood measurements, each sample was collected in a Vacutainer tube (Becton Dickinson, New Jersey, USA) containing potassium EDTA as an anticoagulant. The content was stirred slowly by inverting the tube 8 times. Each tube was tagged, wrapped in clear plastic, settled in a liquid nitrogen tank and preserved in these conditions until arrival at the laboratory.

Laboratory measurements were performed using Pointe Scientific reagents. The reaction was run in Eppendorf tubes and absorbance was read in a microplate lector with a wavelength of 492 nm.

Hb concentrations were measured in automated hematologic Beckman Dickinson Coulter equipment (Fullerton CA, USA) using Pointe Scientific reagents. A fresh blood sample was used as a calibrator.

All measures with the three procedures were obtained from participants on the same day.

### Statistical analysis

Paired t-tests and simple linear regression models with no intercept were estimated for Hemocue and Masimo measurements as predictors of the gold standard measurement which represented the true expected Hb concentration. The regression models were set up to estimate predictability based on the determination coefficient (r^2^). Accuracy was verified against a regression coefficient (β) equal to 1 (indicating no measurement bias). These models can be used to establish a calibration procedure for correcting measurements and obtaining accurate (unbiased) Hb estimates in capillary samples. This is particularly relevant when estimating correspondence between HemoCue, Masimo and venous blood measures.

We used the Concordance Correlation Coefficient (CCC) method [20] to evaluate random (precision) and systematic (accuracy) errors in HemoCue and Masimo Hb measurements, taking the gold standard as reference. CCC combines measures of precision and accuracy to determine how far observed data deviate from the line of perfect concordance (that is, the line at 45° on a square scatter plot). CCC can be expressed either as the product of the Pearson correlation coefficient to indicate precision or as the bias-correction factor to indicate accuracy. We also used the Bland-Altman limits of agreement [21] procedure. Like CCC, it considers random and systematic errors, providing a useful measure for comparing the likely differences between individual results from two methods (device vs. gold standard). The Bland-Altman procedure serves primarily to set the confidence interval when estimating the difference between predictor device and gold standard measurements. As this method considers precision and accuracy differently from the traditional paired t-test, it is useful when the latter fails.

The extent of variability in Masimo Hb measurements was attributable to a combination of biological fluctuation and technical error (or device error not adequately isolated). Finally, in accordance with standard industrial Measurement System Analysis (MSA) procedure, we studied the Masimo measurement system using the three-level Hierarchical Linear Model (HLM). This allowed for estimating variance components and for evaluating variance distribution among devices (upper level), sampled subjects (intermediate level) and three Hb measurements per participant (lower level). Table 1 shows descriptive statistics for the repeated measures and evaluates the differences in means and standard deviations. HLM analysis was not feasible for HemoCue because repeated invasive capillary blood sampling could not be performed on the same subject.

**Table 1:** Descriptive statistics for repeated Masimo Hb measurements.

Measurement	Mean	Std. Dev.	95% CI
1st	12.65	1.21	12.5, 12.8
2nd	12.65	1.15	12.5, 12.8
3rd	12.64	1.03	12.5, 12.8

### Ethical considerations

A written informed consent was obtained from the parents/guardians of participating children after carefully explaining to them the objectives and methods of the study as well as the risks involved. Venous blood samples were taken with the assent of the children and in the presence of their parents.

The Research, Ethics, and Biosecurity Committees of the National Institute of Public Health in Cuernavaca, Mexico, approved all procedures involving human subjects.

## Results

We excluded 11 children form the original sample because of implausible Hb values for children under five years. Table 2 shows the estimated mean of Hb concentrations by equipment for the final sample of 137 children. The means obtained with HemoCue and Masimo were significantly lower (p<0.001) than those of the gold standard (laboratory venous blood measures), with paired estimated differences amounting to -1.51 g/dL and -1.62 g/dL, respectively.

**Table 2:** Mean and confidence intervals of Hb by measuring procedure.

Device	Hb g/dL	95% CI
HemoCue 201^*^	12.7	12.5, 12.9
Masimo Pronto 7^*^	12.6	12.4, 12.8
Gold Standard	14.2	14.0, 14.5

*Significant t-test results in comparison of devices against the Gold Standard (p<0.001).

Using the gold standard as dependent variable and HemoCue results as predicting variable (Table 3), our regression analysis yielded 98.7% predictability, indicating a very high predictive power. However, the regression coefficient was significantly below 1 (β = 0.887; p <0.001), showing a significant downward bias. Similarly, predictability for Masimo was high at 98.6%, but the regression coefficient was significantly below 1 (β = 0.876; p < 0.001).

**Table 3:** Linear Regression models for predicting Hb (Gold Standard) using HemoCue 201 and Masimo Pronto 7 as predictors.

Regression model	β	Test for β = 1 F (1, 136)	P	R-squared
HemoCue 201 vs. Gold Stadard	0.887	168.28	0.000	0.987
Masimo Pronto 7 vs. Gold Stadard	0.876	192.63	0.000	0.986

The CCC for HemoCue measurements was 0.183, (p<0.001), with 0.313 precision (Pearson correlation coefficient) and 0.586 accuracy (Table 4). The Bland-Altman limits of agreement showed a mean difference of -1.51 between HemoCue and gold standard values. While the difference did not sway significantly from zero according to the 95% confidence interval, it did shift to the negative values.

**Table 4:** Concordance Correlation Coefficient and Bland-Altman Limits of Agreement for HemoCue 201 and Masimo Pronto 7 as compared to the Gold Standard.

Device	Concordance Correlation	Pr(r = 0)	Precision	Accuracy
HemoCue 201	0.183	0.000	0.313	0.586
Masimo Pronto 7	0.166	0.000	0.299	0.556
Device vs. Gold Standard	Difference	Std. Dev.	95% Limits Of Agreement	
			(Bland & Altman, 1986)	
HemoCue 201	-1.51	1.58	-4.60	1.58
Masimo Pronto 7	-1.67	1.62	-4.84	1.51

Likewise, the CCC for Masimo was 0.166, (p<0.001), with 0.299 precision and 0.556 accuracy. The Bland-Altman limits of agreement yielded an average difference of -1.62.

The estimated variance components in the three-level Hierarchical Linear Model (Table 5) showed device and subject variances of only 16.3% and 25.8%, respectively, but high within-subject (multiple sampling from the same subject) variance. This proved the largest component of total variance, indicating low reliability for Hb measurements among children in field studies. As shown in Table 1, differences in the Masimo means and standard deviations of repeated measures were similar, thus indicating stability in measures over time.

**Table 5:** Variance components for the Masimo Pronto 7 Hb measurements.

Hierarchical level	Variance component	Percentage
Masimo devices	0.193	16.28%
Subjects	0.322	25.81%
Within subject	0.724	57.91%
Total	1.239	100%

## Discussion

Hb measures are an important determinant both in assessing anemia during fieldwork and in balancing the speed and ease of rapid analysis with the accuracy of traditional laboratory methods. The latter have long involved invasive blood draws followed by sample analysis with laboratory devices such as the CO-Oximeter. Recently, however, invasive and noninvasive Hb measurements are being performed using point-of-care devices.

The objective of our study was to evaluate the accuracy and precision of the HemoCue 201 (HemoCue) and Masimo Pronto 7 (Masimo) devices in measuring Hb during epidemiological studies, having venous blood measures as the gold standard. Our regression models demonstrated that Hemocue and Masimo had similar measurement bias and underestimated Hb concentrations compared to the gold standard (Table 3). By contrast, the Bland-Altman limits of agreement method, which considers an interval of zero as a valid difference in measurements (Table 4), yielded a non-significant bias for both devices. Patel et al. [17] mention that capillary Hb measurements using HemoCue are biased downward with respect to venous measurements. However, other authors having performed a systematic review and meta-analysis of method comparison studies maintain that both Hemocue and Masismo provide an unbiased, pooled estimate of laboratory Hb [22].

In comparing Hb measures from both devices, we found high precision (according to regression predictability) but limited accuracy levels, indicating a positive bias in estimated prevalence of anemia. Nonetheless, this can be corrected by using a bias correction approach based on the inverse of the regression coefficients. CCC analysis showed low accuracy and low precision for both devices.

Our results for Masimo performance match those of other studies. It should be noted, however, that these studies were conducted with adults and in clinical environments [15,16,18,19]. As for HemoCue, previous validation studies on the use of HemoCue and Celldyn 1700 in Mexico have blamed biological variability for the difference between the Hb concentrations in venous and capillary blood measurements. It has been reported that Hb concentration in capillary blood measured with HemoCue provides an adequate assessment of the prevalence of anemia in open populations but may lead to false negative diagnoses in individuals [23]. No data are available in this respect concerning Masimo. We have found, however, that both devices are suitable not only for fieldwork with children from one to five years of age, but also for Hb determination in epidemiological studies and in programs that include nutrition/food evaluations. While other considerations need to be evaluated, we can safely affirm that HemoCue and Masimo face no competition in measurement performance. Furthermore, use of these devices for determining Hb in population studies can clearly lead to improvements in field sampling costs, logistics, feasibility and supply expenses.

This study provides a robust contribution to assessing the comparative use of HemoCue and Masimo devices in epidemiological studies among child populations.

Following are a few practical specifications that Masimo users should bear in mind: Fingers must be free of perspiration and other substances, as this can obstruct measurement readings; they must also be warm, given that cold fingers cause measurement errors. Finally, the Masimo system is highly sensitive to light; therefore, to obtain accurate measurements, the sensor in the device must be completely covered. These considerations underscore the necessity of ensuring proper use of the device during fieldwork and, hence, of providing responsible personnel with sufficient training.

According to Hierarchical Linear Model analysis, within-subject variance proved the largest component of total variance in Masimo measurements (Table 5). This suggests that precision could be improved by enhancing the device, for instance, by adjusting the size of the sensor to children: inaccurate measurements may result when the finger does not fit properly in the sensor.

HemoCue users must also bear in mind several logistic specifications; for instance, excessively hot, cold or wet weather conditions may interfere with performance. Furthermore, the microcuvettes must be stored in a dry place and at a temperature of 15–30°C [24]. Operating temperatures must be closely observed in the field.

Masimo is less invasive than HemoCue, and can therefore be used repeatedly among children under five. The latter has been proven in the field for longer than Masimo, but has the disadvantage of requiring a finger puncture to obtain capillary blood samples.

As regards efficiency and convenience, both methods are recommended for fieldwork in epidemiological studies among populations of all ages. Rapid, accurate and reproducible assessment is a critical screening process and has led to the development of point-of-care methods for noninvasive or minimally invasive Hb determination [17].

In conclusion, HemoCue and Masimo devices produce comparable measurements in children. While HemoCue has been used for longer with positive results in a number of epidemiological surveys, Masimo has the advantage of offering a noninvasive procedure. This reduces the non-response rate of children under five in epidemiological studies. Use of both devices requires adequate training of field workers.

## Abbreviations

Hb: (hemoglobin)
SpO2: (arterial oxygen saturation)
PR: (pulse)
SpHb: (total hemoglobin)
PI: (perfusion index)
